# Detection and analysis of RNA methylation

**DOI:** 10.12688/f1000research.17956.1

**Published:** 2019-04-26

**Authors:** Nigel P. Mongan, Richard D. Emes, Nathan Archer

**Affiliations:** 1 School of Veterinary Medicine and Sciences, University of Nottingham, Sutton Bonington Campus, Loughborough, UK; 2 Department of Pharmacology, Weill Cornell Medical Center, New York, NY, USA; 3 Advanced Data Analysis Centre , University of Nottingham, Sutton Bonington Campus, Loughborough, UK

**Keywords:** mRNA, RNA, RNA methylation, Nucleic Acids, m6A, 5 prime cap, sequencing, epitranscriptome, epitranscriptomics, transcriptome

## Abstract

Our understanding of the expanded genetic alphabet has been growing rapidly over the last two decades, and many of these developments came more than 80 years after the original discovery of a modified guanine in tuberculosis DNA. These new understandings, leading to the field of epigenetics, have led to exciting new fundamental and applied knowledge and to the development of novel classes of drugs exploiting this new biology. The number of methyl modifications to RNA is about seven times greater than those found on DNA, and our ability to interrogate these enigmatic nucleobases has lagged significantly until recent years as an explosion in technologies and understanding has revealed the roles and regulation of RNA methylation in several fundamental and disease-associated biological processes. Here, we outline how the technology has evolved and which strategies are commonly used in the modern epitranscriptomics revolution and give a foundation in the understanding and application of the rich variety of these methods to novel biological questions.

## Introduction

Beyond the basic genetic letters of A, G, C, and U, chemically modified nucleotides can be found in the RNA of all eukaryotes. Though originally characterised in the 1970s ^1– 4^, methyl modifications to messenger RNA (mRNA) have been thrust back into the spotlight of late because of the availability of new technology and understanding which have revealed links with fundamental genetic processes and disease. In DNA, the intensive study of modified nucleotides during the last 20 years of the epigenetics revolution has led to the understanding of the role of DNA modifications in swathes of developmental decisions and disease. The epigenetics revolution has resulted in novel drugs, including DNA methylation inhibitors, bromodomain inhibitors, and histone acetyl transferase inhibitors ^5^.

**Figure 1.**
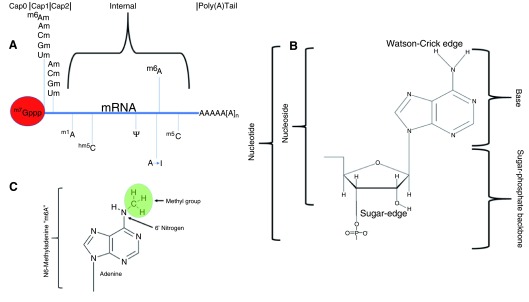
Anatomies of mRNA, nucleotides, and modified nucleotides. Simple diagram of the epitranscriptomic marks found on messenger RNA, given in the context of the 5′ cap and cap-adjacent structures, typical internal modifications, and the poly(A) tail ( **A**). The relevant anatomy of a nucleotide (in this case, adenosine) for the purposes of detection of methylations to the base or ribose sugar is shown in ( **B**). A diagrammatic explanation of the naming convention which describes the base and methylated position to arrive at a simplistic name for the methylated form “m6A” is shown in ( **C**).

Whereas only a handful of methylated nucleotides are found in DNA, dozens are found in RNA, including the 72 methyl-group modifications listed on the MODOMICS database ^6^. Most of these modifications ( Table 1) are found on the nucleoside base rather than the sugar-phosphate backbone ( Figure 1) and only a few of these are found on mRNA, the protein-coding RNA species.

**Table 1.** Methyl modifications to RNA.

Name	Short name	Modified edge	Found on mRNA	Typical location
2’-O-methyladenosine	Am	Sugar	Yes	Cap1, Cap2
2’-O-methylcytidine	Cm	Sugar	Yes	Cap1, Cap2
2’-O-methylguanosine	Gm	Sugar	Yes	Cap1, Cap2
2’-O-methyluridine	Um	Sugar	Yes	Cap1, Cap2
N6,2’-O-dimethyladenosine	m6Am	Sugar and Watson–Crick	Yes	Cap1
1-methyladenosine	m1A	Watson–Crick	Yes	Internal
1-methylguanosine	m1G	Watson–Crick	Yes	Internal
5-methylcytidine	m5C	Watson–Crick	Yes	Internal
N6-methyladenosine	m6A	Watson–Crick	Yes	Internal
7-methylguanosine	m7G	Watson–Crick	Yes	Cap structure (Cap0, Cap1, Cap2)
7-methylguanosine cap (cap 0)	m7Gpp(pN)	Watson–Crick	Yes	Cap structure (Cap0, Cap1, Cap2)
N2,N2,7-trimethylguanosine	m2,2,7G	Watson–Crick	No	Cap structure (snRNA)
N2,N2,7-trimethylguanosine cap (cap TMG)	m2,2,7Gpp(pN)	Watson–Crick	No	Cap structure (snRNA)
N2,N2-dimethylguanosine	m2,2G	Watson–Crick	No	Cap structure (snRNA)
N2,7-dimethylguanosine	m2,7G	Watson–Crick	No	Cap structure (snRNA)
N2,7-dimethylguanosine cap (cap DMG)	m2,7Gpp(pN)	Watson–Crick	No	Cap structure (snRNA)
N4-acetyl-2’-O-methylcytidine	ac4Cm	Sugar and Watson–Crick	No	
5-carboxymethylaminomethyl-2’-O-methyluridine	cmnm5Um	Sugar and Watson–Crick	No	
5-formyl-2’-O-methylcytidine	f5Cm	Sugar and Watson–Crick	No	
1,2’-O-dimethylguanosine	m1Gm	Sugar and Watson–Crick	No	
1,2’-O-dimethylinosine	m1Im	Sugar and Watson–Crick	No	
N2,N2,2’-O-trimethylguanosine	m2,2Gm	Sugar and Watson–Crick	No	
N2,7,2’-O-trimethylguanosine	m2,7Gm	Sugar and Watson–Crick	No	
N2,2’-O-dimethylguanosine	m2Gm	Sugar and Watson–Crick	No	
3,2’-O-dimethyluridine	m3Um	Sugar and Watson–Crick	No	
N4,N4,2’-O-trimethylcytidine	m4,4Cm	Sugar and Watson–Crick	No	
N4,2’-O-dimethylcytidine	m4Cm	Sugar and Watson–Crick	No	
5,2’-O-dimethylcytidine	m5Cm	Sugar and Watson–Crick	No	
5,2’-O-dimethyluridine	m5Um	Sugar and Watson–Crick	No	
N6,N6,2’-O-trimethyladenosine	m6,6Am	Sugar and Watson–Crick	No	
isowyosine	imG2	Watson–Crick	No	
5-carboxymethylaminomethyl-2- thiouridine	cmnm5s2U	Watson–Crick	No	
5-carboxymethylaminomethyl-2-selenouridine	cmnm5se2U	Watson–Crick	No	
5-carboxymethylaminomethyluridine	cmnm5U	Watson–Crick	No	
5-cyanomethyluridine	cnm5U	Watson–Crick	No	
2’-O-methylinosine	Im	Sugar	No	
1-methyl-3-(3-amino-3-carboxypropyl)pseudouridine	m1acp3Y	Watson–Crick	No	
1-methylinosine	m1I	Watson–Crick	No	
1-methylpseudouridine	m1Y	Watson–Crick	No	
2,8-dimethyladenosine	m2,8A	Watson–Crick	No	
2-methyladenosine	m2A	Watson–Crick	No	
N2-methylguanosine	m2G	Watson–Crick	No	
3-methylcytidine	m3C	Watson–Crick	No	
3-methyluridine	m3U	Watson–Crick	No	
3-methylpseudouridine	m3Y	Watson–Crick	No	
N4,N4-dimethylcytidine	m4,4C	Watson–Crick	No	
N4-methylcytidine	m4C	Watson–Crick	No	
5-methyldihydrouridine	m5D	Watson–Crick	No	
5-methyl-2-thiouridine	m5s2U	Watson–Crick	No	
5-methyluridine	m5U	Watson–Crick	No	
N6,N6-dimethyladenosine	m6,6A	Watson–Crick	No	
N6-methyl-N6-threonylcarbamoyladenosine	m6t6A	Watson–Crick	No	
8-methyladenosine	m8A	Watson–Crick	No	
5-methoxycarbonylmethyl-2’-O-methyluridine	mcm5Um	Sugar and Watson–Crick	No	
methylwyosine	mimG	Watson–Crick	No	
5-methylaminomethyl-2-thiouridine	mnm5s2U	Watson–Crick	No	
5-methylaminomethyl-2-selenouridine	mnm5se2U	Watson–Crick	No	
5-methylaminomethyluridine	mnm5U	Watson–Crick	No	
2-methylthio-N6-methyladenosine	ms2m6A	Watson–Crick	No	
5-carbamoylmethyluridine	ncm5U	Watson–Crick	No	
5-carbamoylmethyl-2’-O-methyluridine	ncm5Um	Sugar and Watson–Crick	No	
5-aminomethyl-2-thiouridine	nm5s2U	Watson–Crick	No	
5-aminomethyl-2-selenouridine	nm5se2U	Watson–Crick	No	
5-aminomethyluridine	nm5U	Watson–Crick	No	
2-thio-2’-O-methyluridine	s2Um	Watson–Crick	No	
5-taurinomethyl-2-thiouridine	tm5s2U	Watson–Crick	No	
5-taurinomethyluridine	tm5U	Watson–Crick	No	
2’-O-methylpseudouridine	Ym	Sugar	No	

Initial list taken from MODOMICS ^6^, redundant entries removed (for example, m7G and m7Gppp considered the same for the purpose of this review). Entries are annotated with simple information including: The modified edge of the nucleotide (i.e. whether the methyl group is found on the base itself or the sugar); the species of RNA the modification is normally found on; and the typical position of the modified nucleotide along an mRNA transcript where relevant..

Methyl modifications to RNA form a system of post-transcriptional control mechanisms allowing the fine tuning of gene expression by modifying how the RNA interacts with other components of the cell. Whilst many RNA species are dependent on their methyl groups for their structure and function, the methylation of mRNA and long non-coding RNA appears to possess a level of dynamism that allows the fine tuning of protein-coding genes and cellular processes. Modifications to ribosomal RNA, transfer RNA, and other non-coding RNAs remain active and vibrant fields of research in the area ^7^.

The role of the 5′ Cap and poly(A) tail is centred on the metabolism of the mRNA molecule rather than the encoded information. These structures are not coded for in the DNA but are found on the vast majority of mRNA molecules and have well-established roles in message stabilisation, nuclear export, and translation initiation ^8^; as a result, these structures are not normally considered part of the “epitranscriptome”. Owing to the location of the modifications ( Figure 1) and lack of an antibody and chemical or enzymatic method to readily allow detection, the cap-adjacent Cap1 and Cap2 ribose methylations are less well understood, although they are involved in the innate immune system and the discerning of self from non-self messages ^9^.

Previous works had linked internal N6-methylation of adenosine to fundamental biological processes and developmental decisions ^10^, but in 2011, evidence that the fat mass and obesity-associated protein, FTO, was able to remove the N6 methylation from mRNA was published, linking the modification to those diseases associated with FTO ^11– 14^ and reigniting the interest in mRNA methylation, its detection, and its biological impacts. More recent works have linked the modification to various processes, from the fundamental such as alternative splicing ^15, 16^ to diseases, including cancer ^17^.

Other common epitranscriptomic marks not covered here include the editing of adenosine-to-inosine ^18^ and pseudouridylation ^19^. These modifications do not involve methylation but can affect the metabolism of the RNA without modifying the coding sequence.

These developments led to the field of the “epitranscriptome”, a field of molecular biology that attempts to characterise the causes and effects of modifications to the nucleotides of mRNA that affect not the coding sequence but rather the expression characteristics of the transcript. Understanding these modifications will allow a new level of fine tuning of gene expression and has great potential in fundamental biology, medicine, biotechnology, and crop production ^20^.

It is the availability of technology that has driven these new fundamental understandings as well as the gap in knowledge of the base modifications and ribose modifications. A bisulphite approach used for DNA m5C analysis has also been adapted for RNA ^21^. Similarly, methylations or modifications to the base-pairing “Watson–Crick edge” ( Figure 1) of the nucleotide may sometimes affect the cDNA molecule produced, allowing identification through bioinformatics. Modifications on the “sugar edge” of the RNA molecule do not appear to affect the enzymes involved in reverse transcription under normal conditions. As a result of these limitations, a whole-transcriptome analysis of the 2’-O-ribose methylations on mRNA has not yet been published.

This work focusses on the strategies used to detect and analyse RNA methylation with a particular focus on those modifications found in mRNA. These methods are used to identify methylation targets and locations on the transcript and, alongside other methods, to unravel the downstream effects on the mRNA life cycle.

## From “a fifth nucleotide” to the epitranscriptomics revolution

The first modifications to nucleotides were reported in 1925 ^22^, the first of the “fifth nucleotides” was 5-methyl-cytosine, and early work focussed primarily on the detection and characterisation of the modifications themselves ^23^. It would be several decades before the technological expertise and availability would allow the location and their biological consequences to be understood, leading to the epigenetics revolution of the 2000s ^24^.

The methods used to identify modified nucleotides can sometimes be applied to both DNA and RNA, although owing to the differences in the modified bases, the biochemical structure of RNA, and the fundamental properties of sequencing technologies, the techniques used to study RNA methylation have diverged significantly.

Curiously, there have been several reports of a “fifth nucleotide”, These are usually reporting on different modified nucleotides and has become a hyperbolic term for newly discovered nucleotides in DNA or RNA. As a result, the “fifth nucleotide” can often be ignored – but most likely refers to 5mC in DNA, and m6A in mRNA.

## Common considerations

Detection of the type of methylation found in RNA is preceded by a number of well-established methods for the purification of RNA ^25^. A typical workflow for the purification of mRNA will include the initial isolation of total RNA, followed by the purification of mRNA by their poly(A) tails using oligoDT or by depleting ribosomal RNA through a number of available strategies ^25^. Since mRNA constitutes only 1 to 5% of the total RNA and the other RNA species can be heavily methylated, several rounds of purification are often required for reliable, consistent data from mRNA.

Depending on the downstream protocol, as little as 50 ng of mRNA, as measured by spectrophotometry, may be required. The quality of the RNA is paramount to generating high-quality data and typically is measured by using agarose gel electrophoresis or microfluidic analysis such as the Agilent bioanalyzer (Agilent, Santa Clara, CA, USA). The subsequent characterisation and analysis of the RNA depend on the type of modification ( Figure 1), the abundance of the modification, and any pre-existing knowledge of its sequence context ( Table 2).

**Table 2.** Strategies for the detection of RNA methylation.

Method type	Input quantity	Resolution	Stoichiometry information	Sequence data	Types of modification detected
Radioisotope incorporation	Medium	Low	Medium	Nil	All
Thin-layer chromatography	Low	Low	High	Low	All
Mass spectrometry	High	Medium	Medium	Low	All
Differential enzyme/chemical–RNA interactions	High	High	Low	Maintained	Ribose
Bisulphite RNA sequencing	High	High	Low	Maintained	m5C
Antibody-based sequencing	High	High	Low	Maintained	Base methylations
Big data	None	Low	Very low	Maintained	All

A brief summary of the commonly used methods for detecting modifications to nucleotides in RNA.

## Radioisotope incorporation assays

The earliest works on RNA methylation were carried out by incorporating radioactive isotopes into the RNA. Labelling the methyl donor, s-adenosyl-methionine, with tritium (3H) enables the measurement of methyltransferase activity by scintillation ^26^ as the radioactive methyl group is added onto the nucleoside. Combining this simple assay with various biochemical tools enabled early identification of methyltransferase activity, the initial identification of the m6A writer complex ^27^, and the characterisation of the properties of various enzymes. However, the use of the method is limited where sequence context is desired or in the assaying of *ex vivo* RNA.

## Thin-layer chromatography

A “fifth ribonucleotide” in yeast RNA was first reported in 1957 when Davis and Allen ^28^ published their paper chromatography analysis of previously discarded RNA preparation fractions. These paper chromatography assays evolved over time into two-dimensional thin-layer chromatography (2D-TLC) capable of identifying most of the modified nucleotides present in RNA ^29^. By separating RNA in two dimensions, the nucleotides disperse across the typically cellulose substrate according to their charge and hydrophobicity, two attributes affected by methylation. The pattern then can be imaged with ultraviolet light or by pre-labelling the RNA with radioisotopes. The numerous advances in isolation of RNA, mRNA, and individual mRNA have enabled the adaptation of this method to assay for both internal m6A ^10^ and cap-adjacent modifications Nm and m6Am ^30^, resulting in a sensitive, quantitative and reproducible strategy.

The modern methods require that the RNA be prepared in such a way that the nucleotide of interest is exposed at the 5′ end of the transcript. For example, digesting mRNA with RNAse T1 which, cutting after every guanine, exposes any nucleotide following a guanine, thereby exposing adenosines found within the canonical m6A motif to 5’ end labelling. The 5′-most nucleotide then can be labelled with a radioactive phosphate group from γ-32P-ATP. These then can be separated on 2D-TLC and the resultant spots can be imaged and quantified with autoradiography. Enzymatic decapping and dephosphorylating of the 5′ end of intact mRNA reveal the nucleotide immediately adjacent to the 5′ cap, enabling the assaying of the cap1 structure ^30^.

Where sequence information is already known, site-specific cleavage and radioactive labelling followed by ligation-assisted extraction and thin-layer chromatography (SCARLET) ^31^ can be used to assay the stoichiometry (ratio of modified to unmodified nucleotide) of the methylation at a known site. Here, a 2’-ribose methylated single-stranded DNA (ssDNA) probe complementary to the target site is used to direct RNAse-H–mediated cleavage, exposing the nucleotide for subsequent 2D-TLC analysis.

2D-TLC has been used to identify and characterise the location of various methylated nucleotides and was used in the first mapping of the locations of m6A in bovine prolactin mRNA ^32^. These works localised the m6A modification to the 3′ end of eukaryotic mRNA ^10, 32, 33^, reported on the phenotypic abnormalities resulting from knockouts in a reverse genetics approach ^10, 33^, as well as pointing towards their roles in developmental decisions ^34, 35^.

Owing to the modest quantities of mRNA required and the preservation of stoichiometry information, recent studies continue to use 2D-TLC in combination with other methods to allow a detailed analysis of the methylation states of internal and cap-adjacent nucleotides. However, 2D-TLC–based methods are often avoided because of their use of radioisotope and sensitivity to degradation, they also require a method of revealing the modified nucleotide and thus will miss modifications outside of a known context or sequence, or where it is not possible to reliably expose only the nucleotide of interest, such as the cap2 nucleotide. 2D-TLC provides excellent stoichiometric information but provides only a general transcriptome-wide view of the methylation status and yields little insight into the sequence specificity.

## Mass spectrometry

In combination with separation based on biophysical properties, mass spectrometry (MS) can identify nucleotides by the mass-to-charge ratio in comparison with known standards. In many ways, MS identification is similar in principle to the chromatography-based methods but without the need for radioisotope or specific exposure of the nucleotide of interest for labelling. The method can detect methylation on the sugar and Watson–Crick edges of the nucleotide. MS also enables the profiling of methylations found on a variety of RNA fragments, including the cap2 structures that are typically inaccessible in 2D-TLC. Enhanced by the available sequencing information, MS has been used to map the modifications to transfer RNA (tRNA) ^36^.

Using MS in the analysis of mRNA has been performed to aid in the measurement of Cap1 and Cap2 structures. However, a major drawback of MS-based methods is the extremely large quantities of RNA required and the need for pre-existing sequencing information for the most informative results to be generated.

## Bisulphite sequencing

Sodium bisulphite treatment deaminate cytosine to uracil, resulting in a mutation to thymine during reverse transcription, which then is revealed in the final sequencing dataset. Methylation of the cytosine at the fifth carbon position protects cytosine from this deamination. This strategy has been widely used in the study of DNA methylation to single base-pair resolutions ^37^.

Given the harsh reaction conditions, sequencing bisulphite-treated RNA was largely considered futile; however, adaptations to the protocol have enabled the use of bisulphite sequencing in a whole-transcriptome manner ^38^. For these, a large quantity of RNA is required to be incubated at high temperatures in a buffer containing sodium bisulphite. RNA libraries then can be prepared by using a standard library preparation protocol for fragmented RNA. The identification of m5C sites then can be carried out by identifying events of cytosine conversion to thymidines in the treated sample against an untreated control or by comparison with a reference genomic dataset. Those sites that remain cytosines in the final data were protected by the methylation.

The limiting factor in bisulphite-RNA-sequencing is the initial large quantity of RNA required to compensate for the high losses caused by the bisulphite treatment; at the same time, neighbouring modifications or double-stranded regions may be resistant to bisulphite treatment, especially under the gentler treatment required to maintain the RNA integrity. At the same time, the most common modification to eukaryotic mRNA is m6A rather than m5C.

## Antibody-based sequencing and methods

The first whole-transcriptome maps of the internal m6A methylated nucleotide were published in 2012 ^39, 40^. Although these methods have been enhanced over the last 7 years, the principles have largely remained unchanged. Antibodies are available for most of the base modifications, and strategies to sequence them mostly follow the established “methylated RNA immunoprecipitation sequencing” (or “MeRIP-seq”) ^40^.

The first two methods—named m6A-seq ^39^ and MeRIP-seq ^40^—were designed to detect N6-adenosine methylation in mRNA and long non-coding RNA but can be applied to any modification with available antibodies; following isolation of poly(A) RNA, the RNA is fragmented to 100 base-pair lengths before being immunoprecipitated with an antibody to N6-methyl adenosine (m6A). The precipitated fractions then can be sequenced with a standard RNA-sequencing approach. During the downstream bioinformatics, the fragments are aligned to the transcriptome. Regions that are enriched after immunoprecipitation are assumed to contain a methylated nucleotide.

Although these early methods enabled the transcript-specific identification of methylated mRNAs, they suffer from low resolution of about 100 to 200 nucleotides and require significant quantities of input RNA. Furthermore, if the antibody exhibits non-specific binding (for example, to a poly(A) stretch rather than a modified m6A site), false positives can be generated. To tackle some of these issues, recent methods have included ultraviolet crosslinking—with either an incorporated neighbouring photoactivatable ribonucleotide (PA-m6A-seq ^41^) or any nearby nucleotide (miCLIP ^42^)—in order to induce detectible mutations or truncations during the downstream library preparation.

Antibodies can also be used in more traditional ways such as dot-blots ^43^; this is an inexpensive method to assay for m6A modification but provides less information.

These strategies significantly increase the resolution gained from sequencing the precipitated fractions, although they still do not yield the quantitative stoichiometric information available through chromatography-based methods. Antibodies have been used to generate, for example, transcriptome-wide “MeRIP” maps of m6A ^39, 40^, m1A ^44^ and m5C ^45, 46^ by using this general methodology, although issues surrounding the specificity of the antibodies used continue to be a point of contention ^47^.

## Reverse transcription stops

Owing to their reliance on antibodies or resistance to chemical deamination, many current methods for the analysis of RNA methylation are able to discern only the modifications to the base. To assay the modifications to the sugar-phosphate backbone of RNA or the “sugar edge” of the nucleotide, several methods that also allow sequence information to be maintained have emerged. These methods typically exploit changes to the biochemistry of the phosphate groups surrounding a change to the 2’-O-ribose methylated site.

Methylation at the 2’-O-ribose position confers resistance to alkaline hydrolysis at neighbouring nucleotides; alkali-hydrolysed RNA then can be ligated to an adaptor for reverse transcription and gaps revealed by RNA sequencing. This method has enabled the transcriptome-wide sequencing of ribose methylations in rRNA ^48^. An orthologous method performs reverse transcription under a limiting concentration of dNTPs, causing some reverse transcriptases to stall and terminate cDNA synthesis at the site of 2’-O-ribose methylations. These truncations are then detectable during the downstream bioinformatics, as exploited by RTL-P ^49^. However, this method requires that enough sequence information be maintained on the protected fragments, limiting its use at the ends of the RNA molecules.

## Bioinformatics and processing

The data produced by the strategies described here are complex, resulting in a growing need for robust bioinformatics approaches. Following alignment of the sequencing data to the genome or transcriptome, bioinformatics tools must interpret the peaks (antibody immunoprecipitation), troughs (reverse transcriptase stops), or mutations (deaminations and non-canonical base pairing) in the sequencing data.

A typical bioinformatics workflow for mRNA methylation analysis begins with initial basecalling, adaptor trimming, and demultiplexing of both an untreated and an immunoprecipitated sample for each replicate. Depending on the type of library preparation strategy used, collapsing polymerase chain reaction duplicates by identifying unique molecular barcodes included on the reverse transcription primer in order to increase the accuracy of the methylation quantification may be possible. Next, an alignment tool (for example, HISAT ^50^ or STAR ^51^) that is able to span exon–exon reads (or using a transcriptome index) is used to align the sequencing data to a known genome.

If a mutation is expected where methylation is deposited, the CLIP Tool Kit ^52^ (CTK) CIMS algorithm can be used to call these sites, whilst a truncation site can be called with the CITS algorithm. A number of software packages exist for analysis of CLIP data, such as MACS2 ^53^, and these will work for RNA immunoprecipitated with antibodies to methylated nucleotides. The “peaks” generated in this way are areas of the genome that are enriched in the immunoprecipitated samples and so must contain the methylated nucleotide. If enough fragments are sequenced, locating the precise modified base is possible although this is dependent on the fragment size and sequencing depth of the employed sequencing technology.

Following the association of methylation with a gene’s transcript, further bioinformatics is likely to include motif analysis ^54^ for the identification of sequence contexts of the methylation and functional annotation such as Gene Ontology (GO) enrichment ^55^ in order to identify the enriched biological characteristics of genes. Other bioinformatics and experiments are likely to follow in order to understand the biological consequences of the methylation; these are beyond the scope of the present work but consist largely of methods to assay the metabolism of the RNA ^56^.

## A note on “big data”

Where the methyl group is found at a location normally involved in base pairing (that is, the Watson–Crick edge of the nucleotide), reverse transcriptases are unable to pair the nucleotide with its complement, resulting in a consistent “stop” or mutation during reverse transcription. These artefacts in the sequencing data may present an opportunity to detect possible methylations in publicly available datasets.

The increase in publicly available sequencing data has enabled the development of software such as HAMR ^57^, which was able to discern tRNA modification sites from such public datasets. However, relying on reverse transcriptase dynamics can result in the loss of information about rare methylation events, methylation on rare transcripts, and stoichiometric information and thus is not so readily applicable to the analysis of mRNA.

## The future of RNA methylation detection

Given the variety of methylated nucleotides found in RNA, it is likely to be some time before a single gold standard strategy exists which allows the preservation of all of the desired characterisations, sequence, and stoichiometry information of a given modification. The most likely candidate appears to be nanopore sequencing technology, which uses changes in electrical charge to discern the nucleotides passing through a protein or graphene nanopore in substrate. Since these methods require no conversion to DNA and analyse the nucleotide by how they affect the charge passing through the substrate, nanopore sequencing is also able to distinguish modified bases from their unmodified counterparts ^58^. Whilst this technology is rapidly advancing, the current lack of read depth and requirement for high computing power appear to be the major limiting factors in the more widespread adoption of this technique for analysis of the diverse mammalian transcriptomes.

Given that methylated nucleotides can be used as biomarkers from a number of non-invasive tissues ^59^, understanding their clinical roles is likely to be a major feature of future research. Therefore, the development of new technologies that can quickly identify the types of modifications found in disease is likely to be paramount to the application of epitranscriptomics both to medicine and in industry.

## Summary

Methylation of nucleotides confers several unique properties to RNA during the life cycle of the molecule but also to their fundamental chemistry, which in recent years has been exploited in order to detect them. Owing to the huge variety of methylated nucleotides found in RNA, the epitranscriptomics revolution has lagged behind the epigenetics revolution by some 20 years. However, study of these modifications has been rapidly evolving over the last decade because of the availability of new technology, and curiosity has been ignited by their newfound associations with economically and medically important arenas of biochemistry.

In many ways, the technology remains the limiting factor. Currently, there is no single best way to detect and analyse the variety of structures associated with RNA methylation, often resulting in a need for a combinatorial approach to gain insights into their stoichiometry whilst maintaining sequence information ( Table 2). It is the development of new technologies over the last decade that has allowed the novel insights into fundamental biological processes and disease, and it is the continued development of these technologies that ultimately will lead towards an ability to rapidly detect the earliest signs of the changes to the cellular metabolism that may be indicative of disease. Better technological availability will also enable some of the growing controversies to be addressed, driven largely by the poor transparency of experimental protocols and poor understanding of how best to analyse these novel sequencing datasets.

Deeper understanding of the epitranscriptome and its consequences can only lead to a better ability to control gene expression in desirable ways. The epigenetics revolution is now leading to improved diagnostics and therapeutics for disease, and the fruits of the epitranscriptomics revolution are likely to be as exciting.

## Abbreviations

2D-TLC, two-dimensional thin-layer chromatography; FTO, fat mass and obesity-associated protein; MeRIP-seq, methylated RNA immunoprecipitation sequencing; mRNA, messenger RNA; MS, mass spectrometry
